# The Heat Shock Response Under Natural Conditions in Two Paper Wasp Species

**DOI:** 10.3390/insects16080849

**Published:** 2025-08-16

**Authors:** Astrid Bay Amstrup, Helmut Kovac, Helmut Käfer, Anton Stabentheiner, Jesper Givskov Sørensen

**Affiliations:** 1 Institute of Biology, University of Graz, Universitätsplatz 2, 8010 Graz, Austria; helmut.kaefer@uni-graz.at (H.K.); anton.stabentheiner@uni-graz.at (A.S.); 2 Section for Genetics, Ecology and Evolution, Department of Biology, Aarhus University, Ny Munkegade 114, 8000 Aarhus, Denmark

**Keywords:** heat shock response, heat shock protein, *hsp*, *Polistes*, paper wasp, heat stress, climate

## Abstract

In a warming world, mechanisms, such as the heat shock response, which plays a role in the recovery from or tolerance towards heat stress, are of increasing importance. Certain heat shock protein genes (*hsps*) have previously been shown to be highly inducible in several paper wasp species during high temperature stress under laboratory conditions. Here, we sought to investigate how *hsps* are expressed under natural daily temperature fluctuations by collecting broods from wild nests of two species of paper wasps (*Polistes dominula*, *P. nimpha*). We collected them at 06:00 and 16:00, the expected low and high points in daily *hsp* expression, and measured the expression of three *hsps* (*hsp70*, *hsp83*, *hsc70*). We found that the nest temperature in July could reach temperatures that result in a large upregulation of *hsp* expression in the laboratory. Under natural conditions, however, we found that the induction response was much more moderate and limited to the larvae. The pupae showed no response to the daily fluctuating temperature and had a constant expression similar to that of larvae in the afternoon. The similarities and differences between natural and laboratory *hsp* expressions highlight the importance of field studies, as they add valuable context when interpreting laboratory results.

## Simple Summary

Paper wasps live in a wide variety of thermal environments, and it has previously been shown that different species from different climates use the heat shock response (HSR) differently. The HSR is a cellular stress response, in which the expression of heat shock protein genes (*hsps*) can aid in the recovery from and resistance towards stressors such as heat stress. Much of the available knowledge on the HSR comes from laboratory studies, including all knowledge from paper wasps. In this study, we sought to investigate whether paper wasp broods express *hsps* under natural conditions. We did this by collecting larvae and pupae in the morning and afternoon, which were the expected low and high points of *hsp* expression. While some aspects of *hsp* expression in this field study matched those under laboratory conditions, some dissimilarities emerged. The difference between *hsp* expression in the morning and afternoon was smaller than in laboratory studies, despite afternoon temperatures exceeding the laboratory heat stress temperature in some cases. Moreover, only larvae, and not pupae, showed significant changes in *hsp* expression. These findings highlight the importance of field studies, as they can reveal differences to take into account when applying knowledge from laboratory studies to real world situations.

## 1. Introduction

In small ectotherms, body temperature is highly dependent on the ambient temperature, which means that the ambient temperature is one of the most important environmental influences on a lot of organismal processes [1]. A species’ current distributional range is determined by its thermal preferences and limits [2,3,4]. However, with the increasing average temperatures and the frequency and severity of extreme thermal events caused by climate changes [5,6], the possibility of an organism encountering detrimentally warm thermal conditions in its habitat is increasing.

When encountering challenging thermal conditions, certain behaviors and physiological mechanisms can help the organism cope with the stress experienced [1,7,8,9,10,11]. Heat avoidant behavior can be used to exploit differences in microhabitat temperatures, but the effectivity is highly dependent on the mobility of the organism and the heterogeneity of microhabitats [12,13,14]. Several physiological mechanisms exist that can help an organism cope with heat stress, one of them being the heat shock response (HSR) [15,16,17]. The HSR is made up of the expression of molecular chaperones called heat shock proteins (Hsps), which aid recovery from and increases resistance towards stress [14,15,18,19,20]. The majority of information about the HSR has been obtained from laboratory experiments under controlled conditions (but see, e.g., [21,22]). While this is useful for determining a lot of characteristics about the HSR and individual *hsps* (genes coding for Hsps), studies under natural conditions are necessary to interpret results in an ecological context, as performance in a natural setting can vary significantly from that in a laboratory setting [17,22,23,24,25]. Under natural conditions, parameters, like acclimation to daily temperature fluctuations and multiple stressors and their interactions (temperature, desiccation, starvation, infection, etc.), may influence both basal *hsp* expression and the induction response [15,17,21,22,23,26].

The paper wasp genus (*Polistes*) has a cosmopolitan distribution, which means that its species have managed to adapt to widely different climates [27,28,29]. Previous studies have suggested that adaptation of how the HSR is utilized could be part of the reason why these species are so adept at inhabiting different thermal environments [30,31]. Paper wasps exhibit a social structure where one or, in some species, more reproductive queen(s) and female workers care for the brood in their nest. Part of brood care is managing the nest temperature during the day, as it can increase rapidly due to insolation [9,11,32,33,34]. The adults achieve this temperature management by collecting and depositing water droplets in empty nest cells for evaporative cooling and by fanning their wings [9,11,32,33,34]. This means that the brood is highly dependent on the adults with regards to the temperature experienced, as they themselves have a very limited capacity for behavioral thermoregulation. Therefore, the brood can be exposed to high temperatures when the ambient temperature and/or insolation is high and the thermoregulatory behavior of the adults is not sufficient [9,11,34]. Mechanisms, such as the HSR, can help cope with the high temperature experienced. It has previously been shown in laboratory studies that the HSR is used differently by different paper wasp species depending on the life stage and climate of origin [30,31]. However, it is not known how the HSR is used by paper wasps under natural conditions.

We sought to investigate this in the present study using two species of *Polistes*: *P. dominula* and *P. nimpha*. We chose these two species as they were present in the same habitat and because it has previously been shown that different species of *Polistes* use the HSR in slightly different ways [30]. The experiments were performed with nests located in Gschwendt in Styria, Austria, which is well within the distribution range of both species. We collected samples of larvae and pupae in the morning (06:00) and afternoon (16:00) on warm days, when maximum nest temperatures reached 38–48 °C. The samples were then used to determine the expression of three *hsps*: *hsp70*, *hsp83*, and *hsc70*. Based on this, we sought to determine if the HSR would be induced to help cope with high temperatures experienced under natural conditions and to make a comparison with induction under laboratory conditions. Amstrup et al. [30] showed that induction of these three *hsps* (*hsp70*, *hsp83* and *hsc70*) happens in the laboratory at 45 °C but not at 35 °C, meaning the minimum temperature of induction would be somewhere in between. We hypothesized that we would find higher *hsp* expression in the afternoon compared to the morning, when the nest temperature was higher.

## 2. Materials and Methods

### 2.1. Sample Collection

This study used two species of paper wasps, namely *P. dominula* and *P. nimpha*, from Styria, Austria. For each species, four nests were investigated. The *P. nimpha* nests were all located in the loft of a house attached to the underside of the roof tiles. Two *P. dominula* nests were located under that same roof (nests 1 and 2), while the other two nests were located in a wooden box mounted on a south-facing brick wall. The experiments were conducted on days when the temperature was high, and weather conditions had been warm and dry for a few days. For *P. dominula,* this was 10 July 2023 and 11 July 2023, and for *P. nympha*, it was 12 August 2023.

**Figure 1:**
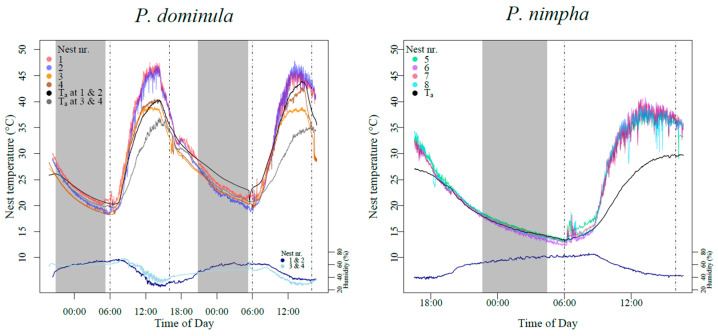
Nest temperatures, ambient temperature (T_a_), and ambient humidity for *P. dominula* (9–11 July 2023) and *P. nimpha* (11–12 August 2023). Grey blocks show the time between sundown and sunup. Punctured lines show sample points. The bottom graphs show concurrently measured ambient humidity, with values displayed on the right, secondary *y*-axis.

We measured temperature directly at the nests (within 3 cm of the nests) and the ambient air with NiCr/Ni thermocouples, as well as humidity of the ambient air (1–2 m from the nests), and stored the data using data loggers (ALMEMO 2690, Ahlborn GmbH, Holzkirchen, Germany). Measurements started in the late afternoon or early evening the day before the experiments to record the overnight temperature and ended after the conclusion of the individual collections. The recorded temperature data can be seen in Figure 1.

At each sampling time, we also took thermograms of the individual nests with a FLIR T650sc camera (resolution 640 × 480 pixels, sensitivity < 20 mK, FLIR Systems Inc., Danderyd, Sweden) to estimate temperature differences across the nests. The pictures were evaluated using FLIR ThermaCam Researcher Pro 2.10 (FLIR Systems Inc., Wilsonville, OR, USA), and data were extracted according to the protocol established in Stabentheiner et al. [9]. From the evaluation, we obtained the mean nest surface temperature (including the surface of any adults present on the nests), standard deviation, and maximum and minimum temperatures. The thermogram nest temperature data can be seen in the Supplementary Material.

For *hsp* analysis, we collected samples at approximately 06:00 in the morning, at the end of the cooler night period, and at 16:00 in the afternoon, 1–2 h after the peak daily temperature. These time points were chosen as they were expected to be the low and the high points of any daily fluctuations in *hsp* expression. For each nest, we aimed at collecting three larvae and three pupae for each collection time point, although that was not always possible due to natural constraints (not enough suitable brood). In total, from the four *P. dominula* nests we collected 10 larvae and 10 pupae in the morning and 12 larvae and 11 pupae in the afternoon. From the four *P. nimpha* nests, we collected 8 larvae and 9 pupae in the morning and 12 larvae and 8 pupae in the afternoon.

To obtain results appropriate for comparison with a previous laboratory study [30], only medium- to large-sized larvae were used (estimated 3rd–5th instar). At each collection point, the samples (single larvae or pupae) were immediately moved to individually marked 2 mL sample tubes and snap frozen in liquid nitrogen. The *P. nimpha* samples were all collected on the same date, while the *P. dominula* samples were collected over two consecutive days with approximately half the samples from every nest collected each day. Following collection, the samples were stored at −80 °C until they were used for qPCR.

### 2.2. qPCR Procedure

Each sample was homogenized using a Qiagen TissueLyser II (QIAGEN GmbH, Hilden, Germany), and RNA was then extracted from the samples with a MicroElute^®^ Total RNA Kit (Omega Bio-tek, Norcross, GA, USA), following the manufacturer’s instructions. Two deviations were made from the instructions: 1. only half the sample volumes were used after the homogenization, as the samples were bigger than intended for the kit, and 2. the RNA was eluted using 100 µL nuclease-free water on the final step. Next, we used a Qubit^TM^ RNA BR Assay Kit and a 157 Qubit^®^ 2.0 Fluorometer (Thermo Fisher Scientific, Waltham, MA, USA) to measure the RNA concentration of the samples and subsequently diluted the samples to an RNA concentration of 3.6 ng/µL. Up until this point, the samples were stored at −80 °C between steps. We then used an Omniscript^®^ RT Kit (QIAGEN GmbH, Hilden, Germany) to synthesize cDNA following the Quick-Start protocol and stored the eluate at −20 °C.

Lastly, we measured the expression of *hsp70*, *hsp83*, and *hsc70* using real-time quantitative PCR (qPCR). The qPCR was run using a Mx3005P Real-Time PCR System (Agilent Technologies, Santa Clara, CA, USA) with a standard two-step thermal profile and a dissociation curve. Brilliant II SYBR^®^ Green qPCR Master Mix (Agilent Technologies, Santa Clara, CA, USA) functioned as dye and ROX as reference dye. As there were too many samples to fit them all into a single qPCR plate, the samples were randomly distributed into two groups. All samples were measured in two technical replicates. In addition to the samples, each qPCR plate also held two types of non-template controls (which were also measured in technical duplicates): one with master mix and nuclease free water replacing the cDNA template and one with the full volume consisting only of the master mix. We used NCBI Primer-Blast [35] to design the primers based in mRNA sequences identified with NCBI GenBank [36]. The primers (Table 1) were ordered from Sigma-Aldrich. As no transcriptome was available for *P. nimpha*, the primers were designed for mRNA sequences for *P. dominula*. The two *Polistes* species used here are closely related [28], and heat shock proteins are generally highly evolutionarily conserved. To validate the identity of the sequences used, we BLASTed each of them against the GenBank database and found that all had more than 90% identity with equivalent genes in two distantly related paper wasp species [35]. Lastly, we examined the amplification plots and dissociation curves and found that amplification commenced similarly in both species and that melting curves confirmed that the amplified sequences were pure and of the same length. Based on this, we are confident that the primers targeted the same gene sequences in both species. A similar approach was used in previous experiments with two other species of *Polistes* closely related to *P. dominula* without any difficulties [30,31]. Furthermore, average amplification efficiencies were close to 1 and similar between the two species for the three genes: *hsp70* (*P. dominula*: 1.02, *P. nimpha*: 0.99), *hsp83* (*P. dominula*: 1.01, *P. nimpha*: 1.04), and *hsc70* (*P. dominula*: 1.02, *P. nimpha*: 0.92).

### 2.3. Data Analysis

An initial visual examination of dissociation curves and amplification plots from the qPCR revealed a few outliers in which it appeared that the amplification process did not work properly. These outliers (two cases of one technical replicate for two samples of *hsp70* and *hsc70*, each) were excluded from the dataset. We used Data Analysis for Real-Time PCR [37] to estimate the relative cDNA content before amplification (R_0_ values). The R_0_ estimates were averaged across technical replicates, except for the samples of which one technical replicate had been excluded. Next, we used NORMA-gene for normalization of the expression data, which provides a target-data driven normalization of R_0_ values among biological replicates and across genes [38].

To analyze the expression data, we created a mixed effects model for each of the three *hsps* using the “nlme” package [39] in R version 4.2.2 [40]:

(1) log (hsp expression)=ToD×species×dev.group+(1|nest)

Time of day (ToD; morning or afternoon), species (*P. dominula* or *P. nimpha*), and developmental group (dev.group; larva or pupa) were fixed effect variables, and the ID number of the nest, from which each sample was collected, was used as a random effect variable. Nest ID was used as a random variable to account for differences between nests caused by e.g., microclimatic differences and genetic relatedness within the nest, as it was not within the scope of this experiment to determine differences within individual nests. Due to the nature of the expression data, the R_0_ data for each *hsp* were log-transformed for the analysis. We had a number of different measures to quantify temperature conditions: min, max, and average for both ambient and nest temperatures. All of them were correlated with ToD, which is why we used this in the analysis, as it simplifies the statistics compared to the others. Furthermore, ToD also covers the differences in humidity, as the humidity was high during the morning collection and low during the afternoon collection, making it strongly negatively correlated with nest temperature. As it was not possible in this investigation to separate the effects of humidity and temperature, using ToD was more appropriate than using nest temperature and humidity as separate factors. We then produced ANOVA tables for the models using anova.lme() from the “nlme” package [39]. Lastly, we used the model to estimate marginal means with the package “emmeans” [41], which were then used to evaluate significant differences in *hsp* expression between developmental groups and time of day in each of the two species using multiple comparisons.

## 3. Results

**Figure 2:**
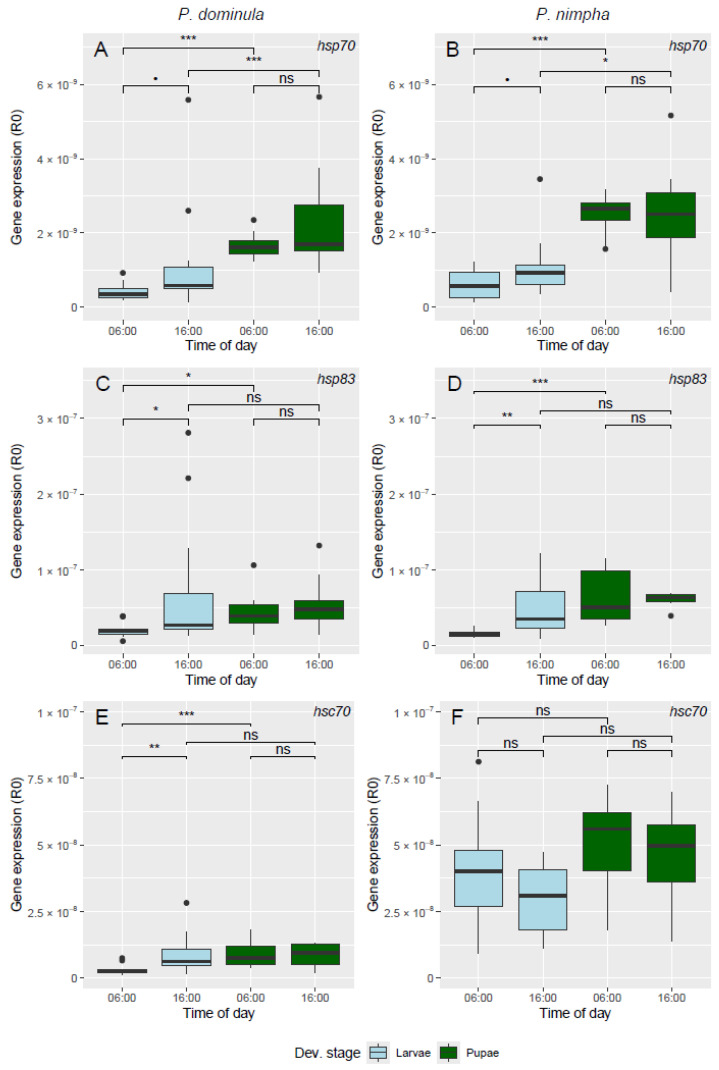
Gene expression in the (cool) morning (06:00) and (warm) afternoon (16:00) of *hsp70* (**A**,**B**), *hsp83* (**C**,**D**), and *hsc70* (**E**,**F**) in *P. dominula* (left column) and *P. nimpha* (right column). Larvae are shown in light blue and pupae in green. Significance levels of comparisons are marked as ns (*p* > 0.1), • (*p* > 0.05), * (*p* < 0.05), ** (*p* < 0.01), and *** (*p* < 0.001). Boxes show upper and lower quantiles, bold lines show the median, whiskers show upper and lower extremes, and circles show outlier data points. For daily temperature course see Figure 1.

An overall analysis showed that the time of day (ToD), developmental group, and the interaction between the two were all significant in explaining variation in *hsp70* expression (Table 2, Figure 2A,B, see Supplementary Material for a depiction of expression levels for individual samples sorted by nest). Neither species nor its interactions had a significant effect on *hsp70* expression. We found that the *hsp70* expression of both species was significantly higher for pupae compared to larvae both in the morning (*P. dominula*: t_(66)_ = 5.46, *p* < 0.0001; *P. nimpha*: t_(66)_ = 5.67, *p* < 0.0001) and in the afternoon (*P. dominula*: t_(66)_ = 4.10, *p* = 0.0007; *P. nimpha*: t_(66)_ = 3.08, *p* = 0.015). For both species we also found that there was no significant difference between *hsp70* expression in the morning and the afternoon for pupae (*P. dominula*: t_(66)_ = 0.83, *p* = 0.84; *P. nimpha*: t_(66)_ = −0.48, *p* = 0.96). There was a trend towards a difference between morning and afternoon in the larvae of both species, although it was not statistically significant (*P. dominula*: t_(66)_ = 2.56, *p* = 0.060; *P. nimpha*: t_(66)_ = 2.37, *p* = 0.093).

When examining the *hsp83* expression (Table 2, Figure 2C,D, see Supplementary Material for a depiction of expression levels for individual samples sorted by nest), we found a significant effect of ToD, developmental group, and the interaction between the two but not of species and its interactions. We found that in the morning, the *hsp83* expression of pupae were significantly higher than that of larvae in both *P. dominula* (t_(66)_ = 2.70, *p* = 0.025) and *P. nimpha* (t_(66)_ = 4.54, *p* = 0.0001). There was no difference between larvae and pupae expression in the afternoon (*P. dominula*: t_(66)_ = 0.65, *p* = 0.92; *P. nimpha*: t_(66)_ = 1.30, *p* = 0.57). The *hsp83* expression of larvae was significantly higher in the afternoon compared to the morning (*P. dominula*: t_(66)_ = 3.02, *p* = 0.019; *P. nimpha*: t_(66)_ = 3.41, *p* = 0.0060). No significant difference between ToD in the pupae were seen (*P. dominula*: t_(66)_ = 0.61, *p* = 0.93; *P. nimpha*: t_(66)_ = 0.041, *p* = 1).

The expression of *hsc70* (Table 2, Figure 2E,F, see Supplementary Material for a depiction of expression levels for individual samples sorted by nest) was significantly affected by species and developmental group but not by ToD. Furthermore, the two-way interactions of ToD × species and ToD × developmental group, as well as the three-way interaction, had significant effects. *hsc70* expression generally appeared to be higher in *P. nimpha* compared to *P. dominula*. We found no significant differences between the experimental groups of *P. nimpha*. When examining the *hsc70* expression of *P. dominula*, we found that the expression of larvae was significantly higher in the afternoon compared to the morning (t_(66)_ = 3.93, *p* = 0.0012), but we found no difference in the pupae (t_(66)_ = −0.24, *p* = 0.99). We also found that, in the morning, pupae had a significantly higher expression than larvae (t_(66)_ = 4.44, *p* = 0.0002), but there was no difference in the afternoon (t_(66)_ = 0.47, *p* = 0.97).

## 4. Discussion

The genes encoding heat shock proteins are known to be highly conserved across the entire tree of life and, as such, have received attention as part of a stress response mechanism in many different ways. Much of the knowledge on heat shock proteins stems from laboratory studies, which can provide a detailed understanding of the proteins and their role in an organism. On the other hand, setups of laboratory studies are typically simplified compared to natural conditions, which can limit their interpretation in an ecologically relevant context. Our results provide insight into the heat shock response (HSR) of paper wasp larvae and pupae under natural conditions and show the usefulness of field studies in bridging the gap between laboratory studies and nature.

We sought to determine whether the HSR would be induced under natural temperature fluctuations as seen in other animals [21,22,26]. To do this, we performed field collections on warm, dry days. At our study site, the nest temperatures in some cases reached ~46–48 °C in July when we collected the samples of *P. dominula*. This exceeds the experimental temperature in a study by Amstrup et al. [30], where a temperature of 45 °C elicited a large upregulation, and is near the upper critical thermal limit [42]. Furthermore, the nests that experienced these high temperatures had a minimum temperature of ~19–20 °C in the morning, meaning that the daily temperature span was bigger than that in the laboratory study by Amstrup et al. [30]. Based on this, we expected to see an upregulation of *hsps* in the afternoon, as the thermal conditions at that time were similar to the laboratory heat stress treatment. This was actually the case in larvae, where the expression of *hsps* was higher in the afternoon compared to the morning (although the difference was not quite significant for *hsp70*). *hsp70* expression was 2.8- and 1.8-fold higher in the afternoon for *P. dominula* and *P. nimpha*, respectively. For *hsp83* the difference was 3.4- and 3.0-fold for the two species, and for *hsc70*, only *P. dominula* showed a significant difference, with the expression being 2.8-fold higher in the afternoon. However, compared to the study by Amstrup et al. [30], where the upregulation after heat stress was in the range of hundred- to several thousand-fold for *hsp70* and ten- to hundred-fold for *hsp83*, the natural induction seen here is much more moderate. A possible reason for the moderate induction response seen in this study could be the thermoregulatory efforts of the adults on the nest. While we measured the temperature directly at the nests, the temperature is not uniform across the entire nest or within the cells, as the adults will periodically fan the nest and deposit water droplets into empty cells for evaporative cooling [9,11,32,33,34]. Thermograms (see Supplementary Material) from the nests show that, at the collection time, the difference between maximum and minimum nest temperature in the afternoon on average was 9.5 °C in *P. dominula* and 7.9 °C in *P. nimpha*. This means that the brood sampled for this study may have been partially or periodically cooled below the measured temperature, which was not the case in the laboratory study, where the adults had been removed from the nests [30]. However, since we only have one thermogram per collection time from each nest, it is not possible for us to determine how different nest microenvironmental temperatures may have influenced *hsp* expression since the microclimatic landscape can change over time depending on where the adults apply their thermoregulatory behavior. The water droplets collected by the adults could also cause an increase in humidity directly at the nest compared to the ambient air. This could mean that the brood in this study experienced less desiccation stress compared to that in Amstrup et al. [30]. However, air turbulence caused by adult wasps landing, taking off, or fanning for nest cooling [11,32] would periodically have reduced any local increase of humidity caused by water evaporation in the nests, which are open to the environment. On its own, desiccation can induce the HSR [43,44], and it can have a negative impact on tolerance towards heat stress [45,46]. However, since we do not have any measures of humidity directly at the nests, it is not possible to determine how it could affect the daily differences in *hsp* expression seen in this study. The relatively smaller induction response in this study compared to the laboratory could also be caused by a different balance between the costs and benefits of *hsp* expression. The expression of *hsps* is costly both in terms of resources and in terms of the disruption of normal development [17,47,48,49]. As natural settings are more complex than a laboratory experiment, the benefits of *hsp* expression might be smaller or the costs more severe, which would result in a smaller induction response. It is also possible that the brood was acclimated to the fluctuating daily temperature, which would decrease the stress experienced and thus result in a smaller stress response. In different species of fruit flies, predictable fluctuations in ambient temperature can, compared to constant temperature, result in increased performance across certain thermal stress resistance traits [50,51,52]. In the Amstrup et al. [30] study, the brood was acclimatized to a constant temperature in the lab for one to four days before the experiments, which could potentially disrupt their natural acclimation, resulting in a larger stress response when encountering the high temperature treatment.

The average temperatures across the nests from the thermograms were generally highly similar to the nest temperature measured by a thermocouple in the morning collections. The afternoon collections had slightly larger differences between the two measurements (up to 5.9 °C in difference) but with an average difference across all nests less than 1 °C. This suggests that while microenvironmental differences exist across the nests, the way we measured nest temperature with thermocouples within 3 cm of the nests is an acceptable measure for average nest temperature.

Our study showed that the expression levels of *hsp83* and *hsc70* were generally higher than that of *hsp70*. This is in line with previous findings in *Polistes* broods [30] and adults [31]. This validates the idea that a high basal expression of these two genes could be contributing to the basal heat stress tolerance of these wasps [30]. It has been shown in honeybees that the expression level of continuously expressed *hsps*, such as *hsp83* and *hsc70*, is strongly associated with adaptation to the thermal environment that a population experiences in nature [53].

Our analysis showed that *P. dominula* and *P. nimpha* did not differ in their expression of either *hsp70* or *hsp83*. On the other hand, *hsc70* expression was significantly higher in *P. nimpha*. Of the three *hsps*, *hsc70* was also the only one in which the pattern between life stages and time of day diverged between the two species. *P. nimpha* showed no significant differences in *hsc70* expression across either time of day or life stage, although there was a tendency towards a lower expression in the afternoon compared to the morning. The high degree of similarity between the two species is contrary to what was previously found in studies of the HSR in *Polistes*, as different species were found to utilize the HSR in different ways, even when they were from the same location [30,31]. It has also been shown in other insects that different species or subspecies from the same or highly similar environments may vary in their use of the HSR [53,54,55]. It is not, however, within the scope of this article to disentangle the reasons for the similarities and differences between the *hsp* expression of the two species, as they were collected approximately one month apart. The expression could be influenced by genetics, temperature conditions, humidity, seasonal conditions, and/or other unknown factors, and so we can only determine that the expression levels of *hsp70* and *hsp83* are similar between the two species and that of *hsc70* is different, without commenting on the exact cause. Furthermore, the HSR has never before been studied in *P. nimpha*, meaning that we cannot compare this species directly with a laboratory study.

We found that, in most cases, the *hsp* expression in the morning was higher in pupae than in larvae, with the only exception being *hsc70* expression in *P. nimpha*. For both species, the *hsp70* expression was higher in the pupae in the afternoon as well. This difference between life stages is similar to what has previously been shown in *Polistes* [30] and could be caused by a difference in CT_max_ (critical thermal maximum), as heat stress tolerance can vary depending on ontogeny [56,57]. A recent study showed that, in species of *Polistes*, the larvae often had a slightly higher CT_max_ compared to pupae [42]. This slight difference would mean that, at any one temperature, the larvae would be experiencing a slightly lower degree of thermal stress compared to the pupae, which would then result in a slightly lower expression of *hsps*. However, the lack of an induction response in pupae in the afternoon seems to go against this theory, as we would then have expected to see a larger induction response in the pupae experiencing the higher degree of thermal stress compared to the larvae [30]. This non-existent induction response in the pupae appears to be the reason why there is no significant difference between pupae and larvae in the afternoon. The lack of a response in the pupae can be explained in a few different ways. First, it is possible that an induction response was hidden by timing. If there was a fast but very short-lived response to the daytime temperature peak [18,58], it could have been gone by the sampling time. This could be resolved in a future study by increasing the number of sampling points throughout the day, which would also increase the understanding of the daily dynamics in *hsp* expression. Second, the costs of expressing *hsps* might outweigh the potential benefits, as discussed above [17,47,48,49]. Third, there is the possibility that other proteins or molecular mechanisms than the three *hsps* studied here are involved. In general, there are many *hsps* available in the HSR, and in, e.g., *Drosophila melanogaster*, genes outside of the HSR seem to be involved in longer-term heat stress management [18,59,60,61]. Lastly, it is possible that instead of doing a daily cycle of *hsp* expression upregulation during the day and downregulation during the night, the pupae simply keep a constant slight elevation of *hsps* compared to the larvae. This would be in line with a study on fruit flies, which showed that acclimation to fluctuating temperatures led to a generally increased CT_max_ with no daily fluctuations [60]. If the pupae, with their slightly lower CT_max_ compared to the larvae [42], have a slightly higher benefit of the constantly higher expression of *hsps*, it could outweigh the costs incurred [17,47,48] that compel the larvae to downregulate their *hsp* expression overnight.

## 5. Conclusions

This field study demonstrates that paper wasps to some degree exhibit changes in *hsp* expression throughout the day under natural conditions, where temperatures at the nests can reach 45+ °C. Only the larvae showed a significant response to daily temperature changes, with the expression being highest in the afternoon. When comparing this field study to a previous laboratory study, we found several similarities: 1. the pupae generally showed a higher expression than the larvae; 2. the expression level of the three *hsps* measured were generally in the same range as their basal expression measured in the laboratory; and 3. the pattern of higher expression of *hsp83* and *hsc70* and lower expression of *hsp70* matches the pattern of basal expression in the laboratory. This study also found that natural *hsp* expression dynamics differed in two main ways from laboratory studies: 1. the pupae showed no significant response to the daily temperature fluctuations, and 2. the induction response in the afternoon was small, with the maximum increase being 3.4-fold of *hsp83*, compared to upregulations of several hundred- or thousand-fold in a laboratory study. This study shows the importance of field studies, as they add ecological relevance to knowledge gained from laboratory studies. In addition, this study suggests that aspects of physiological mechanisms may differ substantially between the laboratory and nature, meaning that a field study can add valuable input towards the interpretation of laboratory results in a real-world context.

## Figures and Tables

**Table 1:** NCBI reference and forward (Fw) and reverse (Rv) primer sequences for the three heat shock proteins investigated.

Target Gene	Target NCBI Reference Sequence	Primer Sequence (5′→3′)
*hsp70*	XM_015333810.1	Fw: ACCCTTGCTGAAACCGAAGA Rv: TGCTCCGCCCTGATGAATTT
*hsp83*	XM_015329297.1	Fw: GCACAGGCACTTCGTGATAC Rv: GCTTCAGCTTTTTGGCGTAGA
*hsc70*	XM_015321430.1	Fw: AGCGGATGGTCAAACCCAAG Rv: CGGGAGGAATTCCGACCAAT

**Table 2:** ANOVA statistics of the expression of *hsp70*, *hsp83*, and *hsc70*. The models used the log-transformed *hsp* R_0_ values as response variables, ToD (time of day), species, and dev.group (developmental group) as fixed effect variables, and the ID of the nest (as seen in Figure 1) as a random effect variable. Displayed is the F-statistics, with numerator degrees of freedom, and *p*-values.

Variable	Gene		
	*hsp70*	*hsp83*	*hsc70*
ToD	F_(1,66)_ = 4.45 *p* = 0.039	F_(1,66)_ = 10.21 *p* = 0.0021	F_(1,66)_ = 1.40 *p* = 0.24
Species	F_(1,6)_ = 0.61 *p* = 0.46	F_(1,6)_ = 0.00 *p* = 0.99	F_(1,6)_ = 49.55 *p* = 0.0004
Dev.group	F_(1,66)_ = 84.14 *p* = < 0.0001	F_(1,66)_ = 21.02 *p* < 0.0001	F_(1,66)_ = 16.69 *p* = 0.0001
ToD × Species	F_(1,66)_ = 0.37 *p* = 0.54	F_(1,66)_ = 0.002 *p* = 0.97	F_(1,66)_ = 5.65 *p* = 0.020
ToD × dev.group	F_(1,66)_ = 4.86 *p* = 0.031	F_(1,66)_ = 8.09 *p* = 0.0059	F_(1,66)_ = 5.00 *p* = 0.029
Species × dev.group	F_(1,66)_ = 0.00 *p* = 0.98	F_(1,66)_ = 1.82 *p* = 0.18	F_(1,66)_ = 0.47 *p* = 0.50
ToD × species × dev.group	F_(1,66)_ = 0.43 *p* = 0.51	F_(1,66)_ = 0.41 *p* = 0.52	F_(1,66)_ = 3.53 *p* = 0.065

## Data Availability

The original contributions presented in this study are included in the Supplementary Material. Further inquiries can be directed to the corresponding authors.

## Supplementary Materials

The following supporting information can be downloaded at: https://www.mdpi.com/article/10.3390/insects16080849/s1, Temperature data; Gene expression data; Figure S1: Gene expression by nest figure; Table S1: Thermogram nest temperatures.

## Abbreviations

The following abbreviations are used in this manuscript:

HSR	Heat shock response
Hsp	Heat shock protein
*hsp*	Gene encoding a heat shock protein
